# Identification of rs7350481 at chromosome 11q23.3 as a novel susceptibility locus for metabolic syndrome in Japanese individuals by an exome-wide association study

**DOI:** 10.18632/oncotarget.16945

**Published:** 2017-04-07

**Authors:** Yoshiji Yamada, Jun Sakuma, Ichiro Takeuchi, Yoshiki Yasukochi, Kimihiko Kato, Mitsutoshi Oguri, Tetsuo Fujimaki, Hideki Horibe, Masaaki Muramatsu, Motoji Sawabe, Yoshinori Fujiwara, Yu Taniguchi, Shuichi Obuchi, Hisashi Kawai, Shoji Shinkai, Seijiro Mori, Tomio Arai, Masashi Tanaka

**Affiliations:** ^1^ Department of Human Functional Genomics, Advanced Science Research Promotion Center, Mie University, Tsu, Japan; ^2^ CREST, Japan Science and Technology Agency, Kawaguchi, Japan; ^3^ Computer Science Department, College of Information Science, University of Tsukuba, Tsukuba, Japan; ^4^ RIKEN Center for Advanced Intelligence Project, Tokyo, Japan; ^5^ Department of Computer Science, Nagoya Institute of Technology, Nagoya, Japan; ^6^ Department of Internal Medicine, Meitoh Hospital, Nagoya, Japan; ^7^ Department of Cardiology, Kasugai Municipal Hospital, Kasugai, Japan; ^8^ Department of Cardiovascular Medicine, Inabe General Hospital, Inabe, Japan; ^9^ Department of Cardiovascular Medicine, Gifu Prefectural Tajimi Hospital, Tajimi, Japan; ^10^ Department of Molecular Epidemiology, Medical Research Institute, Tokyo Medical and Dental University, Tokyo, Japan; ^11^ Section of Molecular Pathology, Graduate School of Health Care Sciences, Tokyo Medical and Dental University, Tokyo, Japan; ^12^ Research Team for Social Participation and Community Health, Tokyo Metropolitan Institute of Gerontology, Tokyo, Japan; ^13^ Research Team for Promoting Support System for Home Care, Tokyo Metropolitan Institute of Gerontology, Tokyo, Japan; ^14^ Research Team for Social Participation and Health Promotion, Tokyo Metropolitan Institute of Gerontology, Tokyo, Japan; ^15^ Center for Promotion of Clinical Investigation, Tokyo Metropolitan Geriatric Hospital, Tokyo, Japan; ^16^ Department of Pathology, Tokyo Metropolitan Geriatric Hospital, Tokyo, Japan; ^17^ Department of Clinical Laboratory, Tokyo Metropolitan Geriatric Hospital, Tokyo, Japan

**Keywords:** body mass index, obesity, metabolic syndrome, genetics, exome-wide association study

## Abstract

We have performed exome-wide association studies to identify genetic variants that influence body mass index or confer susceptibility to obesity or metabolic syndrome in Japanese. The exome-wide association study for body mass index included 12,890 subjects, and those for obesity and metabolic syndrome included 12,968 subjects (3954 individuals with obesity, 9014 controls) and 6817 subjects (3998 individuals with MetS, 2819 controls), respectively. Exome-wide association studies were performed with Illumina HumanExome-12 DNA Analysis BeadChip or Infinium Exome-24 BeadChip arrays. The relation of genotypes of single nucleotide polymorphisms to body mass index was examined by linear regression analysis, and that of allele frequencies of single nucleotide polymorphisms to obesity or metabolic syndrome was evaluated with Fisher's exact test. The exome-wide association studies identified six, 11, and 40 single nucleotide polymorphisms as being significantly associated with body mass index, obesity (*P* <1.21 × 10^−6^), or metabolic syndrome (*P* <1.20 × 10^−6^), respectively. Subsequent multivariable logistic regression analysis with adjustment for age and sex revealed that three and five single nucleotide polymorphisms were related (*P* < 0.05) to obesity or metabolic syndrome, respectively, with one of these latter polymorphisms—rs7350481 (C/T) at chromosome 11q23.3—also being significantly (*P* < 3.13 × 10^−4^) associated with metabolic syndrome. The polymorphism rs7350481 may thus be a novel susceptibility locus for metabolic syndrome in Japanese. In addition, single nucleotide polymorphisms in three genes (*CROT*, *TSC1*, *RIN3*) and at four loci (*ANKK1*, *ZNF804B*, *CSRNP3*, 17p11.2) were implicated as candidate determinants of obesity and metabolic syndrome, respectively.

## INTRODUCTION

Obesity is a major risk factor for coronary artery disease, hypertension, diabetes mellitus, dyslipidemia, and several types of cancer [1, 2]. Epidemiological studies in different populations have recognized a contribution of genetic factors to individual susceptibility to obesity [1, 3], with the heritability of this condition having been estimated at 40% to 70% [4]. Genome-wide association studies (GWASs) and meta-analyses of such studies—most performed with individuals of European descent [5–14] or in East Asian populations [15–17]—have identified >100 loci or genes that influence body mass index (BMI) or confer susceptibility to obesity or adiposity.

Metabolic syndrome (MetS) is also a crucial risk factor for cardiovascular disease, diabetes mellitus [18, 19], and cancer [20, 21]. The heritability of MetS has been estimated to be ~30% [22, 23]. GWASs have implicated various loci or genes in predisposition to MetS or to traits of this syndrome in individuals of European [22, 23] or African [24] ancestry or in Asian Indians [25].

Genetic variants identified in previous GWASs typically have a minor allele frequency (MAF) of ≥5% and a small individual effect size. Given that these common variants explain only a fraction of the heritability, it is expected that low-frequency (0.5% ≤ MAF < 5%) or rare (MAF < 0.5%) variants with larger effect sizes also contribute to the genetic architecture of obesity or MetS [26]. Although several polymorphisms have been found to be significantly associated with BMI in East Asian populations [15–17], genetic variants, including low-frequency and rare variants, that influence BMI or contribute to predisposition to obesity or MetS in Japanese remain to be identified definitively.

We have now performed exome-wide association studies (EWASs) with the use of exome array–based genotyping methods to identify SNPs—in particular, low-frequency or rare coding variants with moderate to large effect sizes—that influence BMI or confer susceptibility to obesity or MetS in the Japanese population. We used Illumina arrays that provide coverage of functional single nucleotide polymorphisms (SNPs) in entire exons including low-frequency and rare variants.

## RESULTS

### EWAS for BMI

We examined the relation of genotypes of 41,327 SNPs that passed quality control to BMI in 12,890 subjects by linear regression analysis. A Manhattan plot of the EWAS is shown in Supplementary Figure 1A. After Bonferroni's correction, six SNPs were significantly [*P* < 1.21 × 10^−6^ (0.05/41,327)] associated with BMI (Table 1).

**Table 1:** The six SNPs significantly (*P* < 1.21 × 10^−^^6^) associated with BMI in the EWAS

Gene	dbSNP	Nucleotide (amino acid) substitution^a^	Chromosome: position	MAF (%)	*P* (genotype)
	rs633715	T/C	1: 177883445	22.0	8.33 × 10^−8^
	rs543874	A/G	1: 177920345	21.9	1.36 × 10^−7^
*FTO*	rs1421085	T/C	16: 53767042	19.1	5.37 × 10^−7^
*FTO*	rs1558902	T/A	16: 53769662	19.1	6.67 × 10^−7^
*FTO*	rs8050136	C/A	16: 53782363	19.2	1.08 × 10^−6^
*FTO*	rs9939609	T/A	16: 53786615	19.3	1.13 × 10^−6^

The relation of genotypes of SNPs to BMI was evaluated by linear regression analysis. ^a^Major allele/minor allele.

### EWAS for obesity

We performed an EWAS for obesity with 12,968 subjects [3954 individuals with obesity (BMI of ≥25 kg/m^2^), 9014 controls (BMI of <25 kg/m^2^)], the characteristics of whom are shown in Table 2. The frequency of men and the prevalence of smoking, hypertension, diabetes mellitus, dyslipidemia, and hyperuricemia as well as BMI, waist circumference, systolic and diastolic blood pressure, fasting plasma glucose level, blood glycosylated hemoglobin (hemoglobin A_1c_) content, and the serum concentrations of triglycerides, low density lipoprotein (LDL)–cholesterol, and uric acid were greater, whereas age and the serum concentration of high density lipoprotein (HDL)-cholesterol were lower, in subjects with obesity than in controls.

**Table 2:** Characteristics of the 12,968 subjects in the EWAS of obesity

Characteristic	Obesity	Controls	*P*
No. of subjects	3954	9014	9014
Age (years)	59.3 ± 12.5	60.8 ± 13.6	<0.0001
Sex (male/female, %)	62.2/37.8	54.3/45.7	<0.0001
BMI (kg/m^2^)	27.2 ± 2.7	21.5 ± 2.1	<0.0001
Waist circumference (cm)	90.3 ± 7.1	77.2 ± 6.2	<0.0001
Current or former smoker (%)	41.0	34.1	<0.0001
Hypertension (%)	64.4	47.9	<0.0001
Systolic blood pressure (mmHg)	135 ± 23	129 ± 24	<0.0001
Diastolic blood pressure (mmHg)	79 ± 13	75 ± 13	<0.0001
Diabetes mellitus (%)	30.5	20.1	<0.0001
Fasting plasma glucose (mmol/L)	6.49 ± 2.56	6.09 ± 2.35	<0.0001
Blood hemoglobin A_1c_ (%)	6.15 ± 1.28	5.80 ± 1.11	<0.0001
Dyslipidemia (%)	75.2	57.5	<0.0001
Serum triglycerides (mmol/L)	1.70 ± 1.14	1.35 ± 0.95	<0.0001
Serum HDL-cholesterol (mmol/L)	1.37 ± 0.38	1.58 ± 0.47	<0.0001
Serum LDL-cholesterol (mmol/L)	3.22 ± 0.87	3.07 ± 0.83	<0.0001
Chronic kidney disease (%)	25.0	23.2	0.0312
Serum creatinine (μmol/L)	77.6 ± 72.6	78.8 ± 87.6	0.4543
Estimated GFR (mL min^−1^ 1.73 m^−2^)	71.8 ± 18.7	72.5 ± 21.2	0.0510
Hyperuricemia (%)	23.7	15.6	<0.0001
Serum uric acid (μmol/L)	348 ± 93	319 ± 94	<0.0001

Quantitative data are means ± SD and were compared between subjects with obesity and controls with the unpaired Student's *t* test. Categorical data were compared between the two groups with Fisher's exact test. Based on Bonferroni's correction, a *P* value of <0.0025 (0.05/20) was considered statistically significant. GFR, glomerular filtration rate.

We examined the relation of allele frequencies of 41,327 SNPs to obesity with Fisher's exact test. A Manhattan plot for the EWAS is shown in Supplementary Figure 1B. After Bonferroni's correction, 11 SNPs were significantly (*P* < 1.21 × 10^−6^) associated with obesity (Table 3). The genotype distributions of these SNPs were in Hardy-Weinberg equilibrium (*P* > 0.001) among both subjects with obesity and controls (Supplementary Table 1).

**Table 3:** The 11 SNPs significantly (*P* < 1.21 × 10^−^^6^) associated with obesity in the EWAS

Gene	dbSNP	Nucleotide (amino acid) substitution^a^	Chromosome: position	MAF (%)	*P* (genotype)	Allele OR
*TRIM36*	rs3749745	G/C (E725Q)	5: 115126517	1.9	5.01 × 10^−38^	0.88
*PDIA5*	rs2292661	C/T (T391M)	3: 123150263	0.7	2.05 × 10^−35^	0.95
*OR10P1*	rs7970885	G/A (V200M)	12: 55637489	19.0	6.82 × 10^−14^	1.01
*CROT*	rs7808249	G/A	7: 87354399	32.0	1.30 × 10^−12^	1.06
	rs2068204	C/T	6: 33090941	37.1	5.02 × 10^−11^	1.02
*DAW1*	rs10191097	T/G	2: 227911955	29.1	7.17 × 10^−11^	0.98
*TRIM40*	rs757259	G/A (E244K)	6: 30147765	16.0	7.21 × 10^−11^	0.98
*TSC1*	rs1076160	A/G	9: 132900647	45.9	4.89 × 10^−10^	0.95
*RIN3*	rs8018360	C/T	14: 92521014	19.9	6.79 × 10^−9^	1.04
*HYOU1*	rs144079825	G/C (E258Q)	11: 119054143	0.1	3.96 × 10^−7^	0.98
*ECE2*	rs145491613	A/G (N388S)	3: 184278550	0.4	9.91 × 10^−7^	0.69

Allele frequencies were analyzed with Fisher's exact test. ^a^Major allele/minor allele. OR, odds ratio.

The relation of the 11 SNPs to obesity was further examined by multivariable logistic regression analysis with adjustment for age and sex (Supplementary Table 2). Three SNPs [rs7808249 (G/A) of *CROT*, rs1076160 (A/G) of *TSC1*, rs8018360 (C/T) of *RIN3*] were related (*P* < 0.05 in at least one genetic model) to obesity, although there was no SNP significantly [*P* < 0.0011 (0.05/44)] associated with this condition (Table 4). The minor *A* and *T* alleles of rs7808249 and rs8018360, respectively, were risk factors for obesity, whereas the minor *G* allele of rs1076160 was protective against this condition.

**Table 4:** Relation of SNPs to obesity as determined by multivariable logistic regression analysis

SNP		Dominant		Recessive		Additive 1		Additive 2	
		*P*	OR (95% CI)	*P*	OR (95% CI)	*P*	OR (95% CI)	*P*	OR (95% CI)
rs7808249	G/A	0.0241	1.09 (1.01–1.18)	0.5172		0.0307	1.09 (1.01–1.18)	0.2039	
rs1076160	A/G	0.2661		0.0340	0.90 (0.82–0.99)	0.6676		0.0410	0.89 (0.80–0.99)
rs8018360	C/T	0.6700		0.0339	1.23 (1.02–1.47)	0.8658		0.0378	1.22 (1.01–1.47)

Multivariable logistic regression analysis was performed with adjustment for age and sex. Based on Bonferroni's correction, a *P* value of <0.0011 (0.05/44) was considered statistically significant. OR, odds ratio; CI, confidence interval.

### Relation of nine SNPs to BMI

We examined the relation of genotypes for nine SNPs to BMI by one-way analysis of variance (ANOVA). The six SNPs (rs633715, rs543874, rs1421085, rs1558902, rs8050136, rs9939609) identified in the EWAS of BMI were all significantly [*P* < 0.0056 (0.05/9)] associated with BMI. Among the three SNPs found to be related to obesity, rs7808249 and rs1076160 were related (*P* < 0.05) to BMI, whereas rs8018360 was not (Table 5).

**Table 5:** Relation of SNPs identified in the present study to BMI

SNP	BMI (kg/m^2^)				P
Associated with BMI					
rs633715	T/C	*TT*	*TC*	*CC*	
		23.1 ± 3.4	23.5 ± 3.6	23.5 ± 3.6	**5.38 × 10^−8^**
rs543874	A/G	*AA*	*AG*	*GG*	
		23.1 ± 3.4	23.5 ± 3.5	23.5 ± 3.6	**9.52 × 10^−8^**
rs1421085	T/C	*TT*	*TC*	*CC*	
		23.2 ± 3.4	23.4 ± 3.5	23.7 ± 3.8	**3.49 × 10^−6^**
rs1558902	T/A	*TT*	*TA*	*AA*	
		23.2 ± 3.4	23.4 ± 3.5	23.7 ± 3.8	**4.28 × 10^−6^**
rs8050136	C/A	*CC*	*CA*	*AA*	
		23.2 ± 3.4	23.4 ± 3.5	23.7 ± 3.8	**6.85 × 10^−6^**
rs9939609	T/A	*TT*	*TA*	*AA*	
		23.2 ± 3.4	23.4 ± 3.5	23.7 ± 3.8	**7.16 × 10^−6^**
Associated with obesity					
rs7808249	G/A	*GG*	*GA*	*AA*	
		23.2 ± 3.4	23.3 ± 3.5	23.3 ± 3.5	0.0266
rs1076160	A/G	*AA*	*AG*	*GG*	
		23.3 ± 3.5	23.3 ± 3.5	23.1 ± 3.4	0.0424
rs8018360	C/T	*CC*	*CT*	*TT*	
		23.3 ± 3.4	23.2 ± 3.5	23.4 ± 3.4	0.6303

Data were compared among genotypes by one-way ANOVA. Based on Bonferroni's correction, *P* values of <0.0056 (0.05/9) were considered statistically significant and are shown in bold.

### EWAS for MetS

We performed an EWAS of MetS with 6817 subjects [3998 individuals with MetS (three or more of the five components of MetS), 2819 controls (none of the five components of MetS)], the characteristics of whom are shown in Table 6. Age, the frequency of men, BMI, waist circumference, systolic and diastolic blood pressure, fasting plasma glucose level, blood hemoglobin A_1c_ content, and the serum concentrations of triglycerides, LDL-cholesterol, creatinine, and uric acid were greater, whereas the serum concentration of HDL-cholesterol and estimated glomerular filtration rate were lower, in subjects with MetS than in controls.

**Table 6:** Characteristics of the 6817 subjects in the EWAS for MetS

Characteristic	MetS	Controls	*P*
No. of subjects	3998	2819	
Age (years)	62.2 ± 11.5	54.1 ± 14.1	<0.0001
Sex (male/female, %)	60.0/40.0	44.7/55.3	<0.0001
BMI (kg/m^2^)	25.5 ± 3.6	21.0 ± 2.3	<0.0001
Waist circumference (cm)	87.0 ± 8.7	74.6 ± 6.2	<0.0001
Current or former smoker (%)	38.2	35.1	0.0111
Systolic blood pressure (mmHg)	141 ± 23	111 ± 11	<0.0001
Diastolic blood pressure (mmHg)	81 ± 14	68 ± 9	<0.0001
Fasting plasma glucose (mmol/L)	7.14 ± 2.93	4.97 ± 0.40	<0.0001
Blood hemoglobin A_1c_ (%)	6.38 ± 1.41	5.41 ± 0.41	<0.0001
Serum triglycerides (mmol/L)	2.08 ± 1.32	0.89 ± 0.32	<0.0001
Serum HDL-cholesterol (mmol/L)	1.27 ± 0.37	1.81 ± 0.43	<0.0001
Serum LDL-cholesterol (mmol/L)	3.24 ± 0.93	3.00 ± 0.74	<0.0001
Serum creatinine (μmol/L)	83.5 ± 95.7	69.0 ± 64.0	<0.0001
Estimated GFR (mL min^−1^ 1.73 m^−2^)	67.0 ± 22.1	77.7 ± 17.7	<0.0001
Serum uric acid (μmol/L)	350 ± 95	297 ± 83	<0.0001

Quantitative data are means ± SD and were compared between subjects with MetS and controls with the unpaired Student's *t* test. Categorical data were compared between the two groups with Fisher's exact test. Based on Bonferroni's correction, a *P* value of <0.0033 (0.05/15) was considered statistically significant.

We examined the relation of allele frequencies of 41,675 SNPs that passed quality control to MetS with Fisher's exact test. A Manhattan plot of the EWAS is shown in Supplementary Figure 1C. After Bonferroni's correction, 40 SNPs were significantly [*P* < 1.20 × 10^−6^ (0.05/41,675)] associated with MetS (Table 7). The genotype distributions of these SNPs were in Hardy-Weinberg equilibrium (*P* > 0.001) among both subjects with MetS and controls (Supplementary Table 3).

**Table 7:** The 40 SNPs significantly (*P* < 1.20 × 10^−^^6^) associated with MetS in the EWAS

Gene	dbSNP	Nucleotide (amino acid) substitution^a^	Chromosome: position	MAF (%)	*P* (allele)	Allele OR
*SLC4A5*	rs10177833	A/C	2: 74230591	46.8	2.51 × 10^−89^	0.98
*TAP2*	rs2071544	A/G	6: 32838344	48.7	2.85 × 10^−76^	1.02
*MYPN*	rs7916821	G/A (S707N)	10: 68174212	28.4	2.78 × 10^−68^	0.96
*PSMD9*	rs14259	A/G (E92G)	12: 121915890	46.3	8.72 × 10^−63^	1.00
*ANKK1*	rs1800497	G/A (E713K)	11: 113400106	37.1	9.27 × 10^−61^	1.02
*ZNF700*	rs75607624	T/G (F290C)	19: 11948884	3.7	1.32 × 10^−53^	0.97
*PLEKHG1*	rs17348890	A/G	6: 150839882	6.6	1.42 × 10^−52^	1.02
*DEPDC7*	rs34161108	G/A (A192T)	11: 33027795	6.2	3.94 × 10^−51^	1.02
*ARHGEF28*	rs536568	A/C	5: 73935841	45.8	9.67 × 10^−44^	0.99
*IGSF22*	rs7125943	C/T (V559M)	11: 18714400	18.6	7.53 × 10^−40^	1.03
*PKD1L1*	rs147417448	T/A (N1607K)	7: 47854920	0.4	1.87 × 10^−35^	0.83
*TICRR*	rs79501973	G/A (V1373I)	15: 89624427	14.7	8.41 × 10^−33^	0.98
*GBF1*	rs143872476	T/C (M240T)	10: 102358118	0.2	2.59 × 10^−31^	1.85
*ZNF804B*	rs1406503	C/G	7: 88931564	5.2	3.38 × 10^−24^	0.84
*GON4L*	rs183379906	C/G (D1943E)	1: 155753217	0.3	6.69 × 10^−23^	1.00
	rs962040	A/G	8: 15454368	30.1	5.33 × 10^−21^	0.98
	rs365488	C/T	6: 29550181	48.8	7.69 × 10^−21^	1.01
*CATSPERD*	rs73544757	T/C (V95A)	19: 5733863	0.3	2.11 × 10^−20^	0.96
	rs1507493	A/G	4: 147108517	26.7	2.03 × 10^−17^	1.03
*MCEE*	rs6748672	C/A (R104L)	2: 71124273	24.2	4.15 × 10^−17^	0.98
*MAP1A*	rs3803335	G/A (R1185H)	15: 43525027	1.6	8.61 × 10^−17^	1.08
*NXPE2*	rs11215158	T/C (V103A)	11: 114698220	16.0	1.82 × 10^−16^	1.06
*TRAT1*	rs79029897	G/A (R179H)	3: 108853852	0.7	1.88 × 10^−16^	1.18
*CCDC13*	rs75893579	C/G (R153G)	3: 42752631	3.9	3.13 × 10^−16^	0.87
*OR10W1*	rs56302613	C/G (S245C)	11: 58267125	3.1	9.91 × 10^−15^	0.85
	rs3135365	T/G	6: 32421478	18.9	2.71 × 10^−14^	0.93
*ACAD10*	rs192237004	A/G (Y578C)	12: 111744661	0.4	5.83 × 10^−12^	1.07
*CSRNP3*	rs1007732	G/T	2: 165602788	34.0	8.13 × 10^−12^	0.92
*TEFM*	rs2433	T/C (I348V)	17: 30899210	18.1	4.20 × 10^−8^	1.03
*B4GALNT2*	rs7224888	T/C (C380R)	17: 49168801	3.5	6.94 × 10^−8^	1.05
*BCAS3*	rs2643103	G/A (S87N)	17: 60709264	12.5	8.49 × 10^−8^	1.02
*PPARGC1B*	rs143268818	C/T	5: 149845772	0.6	8.50 × 10^−8^	0.84
	rs56150213	G/A	17: 19761005	49.4	1.43 × 10^−7^	1.08
*ZNF597*	rs140727539	C/T (E315K)	16: 3436756	0.1	1.45 × 10^−7^	1.01
*GPR179*	rs201149338	C/G (Q1461E)	17: 38329188	3.1	1.51 × 10^−7^	0.89
*TENM2*	rs9313396	T/G	5: 168072827	50.0	2.45 × 10^−7^	1.02
*PDP2*	rs141108875	C/T (R473W)	16: 66885701	0.5	2.69 × 10^−7^	1.50
*SLC15A1*	rs12853441	C/A	13: 98725694	49.1	2.99 × 10^−7^	0.96
	rs7350481	C/T	11: 116715567	27.7	7.43 × 10^−7^	1.21
	rs9500989	C/G	6: 29802381	0.8	7.76 × 10^−7^	1.06

Allele frequencies were analyzed with Fisher's exact test. ^a^Major allele/minor allele. OR, odds ratio.

The relation of the 40 SNPs to MetS was further examined by multivariable logistic regression analysis with adjustment for age and sex (Supplementary Table 4). Five SNPs (rs1800497, rs1406503, rs1007732, rs56150213, rs7350481) were related (*P* < 0.05 in at least one genetic model) to MetS. Among these SNPs, rs7350481 (C/T) at chromosomal region 11q23.3 was also significantly [*P* < 3.13 × 10^−4^ (0.05/160)] associated with MetS, with the minor *T* allele being a risk factor for this condition (Table 8).

**Table 8:** Relation of SNPs to MetS as determined by multivariable logistic regression analysis

SNP		Dominant		Recessive		Additive 1		Additive 2	
		*P*	OR (95% CI)	*P*	OR (95% CI)	*P*	OR (95% CI)	*P*	OR (95% CI)
rs1800497	G/A (E713K)	0.8813		0.0227	1.19 (1.02–1.38)	0.5289		0.0627	
rs1406503	C/G	0.0212	0.82 (0.69–0.97)	0.0597		0.0477	0.84 (0.70–0.99)	0.0545	
rs1007732	G/T	0.0344	0.89 (0.81–0.99)	0.1245		0.0861		0.0435	0.84 (0.71–0.99)
rs56150213	G/A	0.0094	1.17 (1.04–1.31)	0.4890		0.0121	1.17 (1.04–1.33)	0.0478	1.16 (1.00–1.34)
rs7350481	C/T	**7.99 × 10^−5^**	1.23 (1.11–1.36)	0.0018	1.35 (1.12–1.63)	0.0018	1.19 (1.07–1.32)	0.0001	1.45 (1.20–1.77)

Multivariable logistic regression analysis was performed with adjustment for age and sex. Based on Bonferroni's correction, *P* values of <3.13 × 10^−4^ (0.05/160) were considered statistically significant and are shown in bold.

### Relation of five SNPs to components of MetS

We examined the relation of the five identified SNPs to the components of MetS—waist circumference, serum concentrations of triglycerides and HDL-cholesterol, blood pressure, and fasting plasma glucose level—in the 6817 subjects by one-way ANOVA (Table 9). The SNP rs7350481 (C/T) was significantly [*P* < 0.0013 (0.05/40)] associated with the serum concentrations of triglycerides and HDL-cholesterol (for men), whereas the other four SNPs were not associated with any of these parameters.

**Table 9:** Relation of SNPs identified in the present study to each component of MetS

SNP		Waist circumference		Serum triglycerides	Serum HDL-cholesterol		Systolic BP	Diastolic BP	Fasting plasma glucose
		Men	Women	Men	Women				
rs1800497	G/A (E713K)	0.6493	0.0334	0.0662	0.0142	0.3710	0.0874	0.4210	0.0667
rs1406503	C/G	0.6225	0.7892	0.4958	0.3470	0.9441	0.3309	0.3962	0.4527
rs1007732	G/T	0.9507	0.0375	0.2333	0.0145	0.0321	0.1330	0.1602	0.3483
rs56150213	G/A	0.9135	0.1495	0.1220	0.5452	0.8970	0.7663	0.4566	0.4820
rs7350481	C/T	0.9403	0.7694	**1.72 × 10^−18^**	**0.0002**	0.0054	0.1548	0.3894	0.0060

Data are *P* values for comparisons among genotypes by one-way ANOVA. Based on Bonferroni's correction, *P* values of <0.0013 (0.05/40) were considered statistically significant and are shown in bold. BP, blood pressure.

### Relation of SNPs identified in the present study to phenotypes examined in previous GWASs

We examined the genes, chromosomal loci, and SNPs identified in the present study to obesity- or MetS-related phenotypes previously investigated in GWASs deposited in a public database [GWAS Catalog (http://www.ebi.ac.uk/gwas)]. In the case of our BMI and obesity studies, chromosomal region 1q25 was previously shown to be related to BMI [7, 8, 12], obesity [7], and body fat percentage [14], whereas *FTO* was previously identified as a genetic determinant of BMI [6, 12, 27], obesity [7, 28], body fat percentage [14], adiposity [29], and circulating leptin level [30]. The remaining three genes (*CROT*, *TSC1*, *RIN3*) have not been previously associated with BMI or obesity (Supplementary Table 5). In the case of our MetS study, the five identified genes or loci (*ANKK1*, *ZNF804B*, *CSRNP3*, 17p11.2, 11q23.3) have not been previously identified as susceptibility loci for MetS, although *ZNF804B* and chromosomal region 11q23.3 were found to be related to BMI in women [31] or serum concentrations of triglycerides and HDL-cholesterol [32, 33], respectively (Supplementary Table 5).

## DISCUSSION

We have now shown that two SNPs [rs633715 (T/C), rs543874 (A/G)] at chromosomal region 1q25 and four SNPs [rs1421085 (T/C), rs1558902 (T/A), rs8050136 (C/A), rs9939609 (T/A)] of *FTO* were significantly associated with BMI. Chromosomal region 1q25 and *FTO* were previously identified as susceptibility loci for BMI and obesity [6–8, 12, 27, 28]. We have also identified three obesity-related genes (*CROT*, *TSC1*, *RIN3*) that have not previously been implicated as determinants of BMI or obesity. In addition, we identified rs7350481 (C/T) at 11q23.3 as a new susceptibility locus for MetS, with SNPs in *ANKK1*, *ZNF804B*, and *CSRNP3* as well as at 17p11.2 also being implicated as candidate susceptibility loci for MetS.

### SNPs associated with obesity

The carnitine O-octanoyltransferase gene (*CROT*) is located at chromosome 7q21.12 (NCBI Gene, https://www.ncbi.nlm.nih.gov/gene) and is expressed in various tissues and organs including adipose tissue (The Human Protein Atlas, http://www.proteinatlas.org). The CROT protein is a member of the carnitine acyltransferase family and converts 4,8-dimethylnonanoyl-CoA to its corresponding carnitine ester. CROT activates lipid metabolism by promoting the β-oxidation of fatty acids [34]. We have now shown that rs7808249 (G/A) of *CROT* was related to obesity with the minor *A* allele representing a risk factor for this condition. This association of *CROT* with obesity may be attributable to the role of this gene in lipid metabolism, although the molecular mechanism remains unclear.

The tuberous sclerosis 1 gene (*TSC1*) is located at chromosome 9q34.13 (NCBI Gene) and is widely expressed including in adipose tissue (The Human Protein Atlas). *TSC1* and *TSC2* encode proteins that form a complex with each other that functions as an inhibitor of mechanistic target of rapamycin (mTOR) signaling [35, 36]. Deletion, nonsense, or missense mutations of *TSC1* and *TSC2* lead to loss of function of the TSC1-TSC2 complex and constitutive activation of mTOR signaling. Such mutations of *TSC1* or *TSC2* cause tuberous sclerosis, a multisystem disorder associated with tumor formation in the brain, heart, lung, kidney, or eye [37, 38]. We have now shown that rs1076160 (A/G) of *TSC1* was related to obesity, with the minor *G* allele being protective against this condition. The association of *TSC1* with obesity might be attributable to the effect of this gene on protein synthesis and cell growth, although the underlying mechanism remains unknown.

The Ras and Rab interactor 3 gene (*RIN3*) is located at chromosome 14q32.12 (NCBI Gene) and is expressed in many tissues including adipose tissue (The Human Protein Atlas). The RIN3 protein is a member of the RIN family of Ras effectors [39] and activates Rab5 in human mast cells, leading to pathological conditions associated with mast cell–mediated chronic inflammation and mast cell hyperproliferation [40]. A GWAS showed that rs10498635 of *RIN3* was associated with Paget's disease of bone [41]. Obesity is closely linked to chronic inflammation in adipose tissue [42–45]. We have now shown that rs8018360 (C/T) of *RIN3* was related to obesity, with the minor *T* allele representing a risk factor for this condition. Although the mechanism underpinning this association remains to be determined, it may be attributable to an effect of *RIN3* on inflammation in adipose tissue.

### SNPs associated with MetS

We have now identified rs7350481 (C/T) at chromosomal region 11q23.3 as a new susceptibility locus for MetS in Japanese. Chromosome 11q23.3 has previously been shown to be related to serum concentrations of triglycerides and HDL-cholesterol in Japanese and Mexican populations [32, 33]. In our study, rs7350481 was also significantly associated with the serum concentrations of triglycerides (*P* = 1.72 × 10^−18^) and of HDL-cholesterol in men (*P* = 0.0002). It was also related to the serum HDL-cholesterol concentration in women (*P* = 0.0054) and to the fasting plasma glucose level (*P* = 0.0060). The association of rs7350481 (C/T) at 11q23.3 with MetS may thus be attributable to the effect of this SNP on lipid and glucose metabolism, although the underlying mechanism remains to be elucidated.

The ankyrin repeat and kinase domain containing 1 gene (*ANKK1*) is located at chromosome 11q23.2 (NCBI Gene) and is expressed ubiquitously (The Human Protein Atlas). *ANKK1* is closely linked to the D2 dopamine receptor gene, and rs1800497 of *ANKK1* has previously been associated with both addictive disorders [46] and obesity [47, 48]. Moreover, obese individuals manifest fewer D2 receptors in the striatum compared with lean ones [48, 49]. We have now shown that rs1800497 [G/A (E713K)] of *ANKK1* was related to MetS, with the minor *A* allele representing a risk factor for this condition. This association of *ANKK1* with MetS may be attributable to an effect of this gene on obesity.

The zinc finger protein 804B gene (*ZNF804B*) is located at chromosome 7q21.13 (NCBI Gene) and is expressed at a high level in the thyroid gland (The Human Protein Atlas). The rs1406503 (C/G) SNP of *ZNF804B* has previously been shown to be related to BMI in Filipino women [31]. We have shown that this SNP was related to MetS in Japanese, with the minor *G* allele being protective against this condition. It is possible that the association *ZNF804B* with MetS is attributable to the effect of this gene on BMI.

The cysteine and serine rich nuclear protein 3 gene (*CSRNP3*) is located at chromosome 2q24.3 (NCBI Gene) and is expressed ubiquitously (The Human Protein Atlas). The three members of the CSRNP family of nuclear proteins (CSRNP1, -2, and -3) share conserved regions including cysteine- and serine-rich regions and a basic domain, possess a transcriptional activation domain, and bind to the DNA sequence motif AGAGTG [50]. We have now shown that rs1007732 (G/T) of *CSRNP3* was related to MetS, with the minor *T* allele being protective against this condition, although the molecular mechanism underlying this association remains unclear.

Chromosome 17p11.2 has been shown to be related to longevity [51] and glomerular filtration rate [52], but it has not been previously found to be related to MetS. We have now shown that rs56150213 (G/A) at 17p11.2 was related to MetS, with the minor *A* allele representing a risk factor for this condition, although the functional relevance of this association remains unknown.

### General considerations

In a previous GWAS [15] and meta-analyses of GWASs [16, 17] for BMI in East Asian populations, the MAF and effect size (explained variance in BMI) of identified SNPs ranged from 3% to 50% and from 0.02% to 0.20%, respectively. In the case of SNPs of *FTO*, the MAF and effect size of rs12149832 were 20% and 0.20%, respectively [15]; those of rs17817449 were 17% and 0.18%, respectively [16]; and those of rs1558902 were 15% and 0.15%, respectively [17]. In our study, we identified six SNPs associated with BMI, with the MAF and effect size (percentage difference in BMI among genotypes) of rs633715 and rs543874 at 1q25 as well as of rs1421085, rs1558902, rs8050136, and rs9939609 of *FTO* being 22.0% and 1.7%, 21.9% and 1.7%, 19.1% and 2.1%, 19.1% and 2.1%, 19.2% and 2.1%, and 19.3% and 2.1%, respectively. These SNPs were thus common variants with a moderate effect size. For SNPs related to obesity in the present study, the MAF and allele odds ratio for obesity of rs7808249, rs1076160, and rs8018360 were 32.0% and 1.06, 45.9% and 0.95, and 19.9% and 1.04, respectively. These SNPs were thus common variants with a small effect size. The MAF and allele odds ratio for MetS for rs7350481 identified in the present study were 27.7% and 1.21, respectively, revealing this SNP to be a common variant with a moderate effect size. For the four additional SNPs related to MetS in our study, the MAF and allele odds ratio for MetS of rs1800497, rs1406503, rs1007732, and rs56150213 were 37.1% and 1.02, 5.2% and 0.84, 34.0% and 0.92, and 49.4% and 1.08, respectively. These SNPs were thus common variants with a small effect size.

There are several limitations to the present study. (i) There were significant differences in age and sex between subjects with obesity or MetS and corresponding controls, which may be caused by selection bias of study subjects. (ii) Given that our results were not replicated, they will require validation in other populations. (iii) It is possible that SNPs identified in the present study are in linkage disequilibrium with other polymorphisms in nearby genes that are actually responsible for the observed associations. (iv) Three SNPs associated with obesity were not significantly related to BMI, and four SNPs associated with MetS were not related to components of this syndrome. The discrepancy in the obesity study might be attributable to substantial percentages of subjects with BMI close to 25 kg/m^2^. The discrepancy in the MetS study might be attributable to the effects of medical treatment for dyslipidemia, hypertension or diabetes mellitus in most subjects with MetS. A lack of relation to waist circumference might be due to substantial percentages of subjects with waist circumference close to cut-off values. (v) Information of treatment such as medication and duration of treatment was not available in the present study. (vi) The functional relevance of the identified SNPs to BMI or the pathogenesis of obesity or MetS remains to be determined.

In conclusion, we have identified rs7350481 (C/T) at 11q23.3 as a novel susceptibility locus for MetS. We also identified three genes (*CROT*, *TSC1*, *RIN3*) as new candidate susceptibility loci for obesity as well as three genes (*ANKK1*, *ZNF804B*, *CSRNP3*) and chromosome 17p11.2 as new candidate loci for MetS. Examination of genotypes for the identified SNPs may prove informative for assessment of the genetic risk for obesity or MetS in Japanese.

## MATERIALS AND METHODS

### Study subjects

For the BMI, obesity, and MetS studies, 12,890, 12,968, or 6817 subjects, respectively, were examined. The subjects were recruited from individuals as previously described [53].

On the basis of the recent recognition of a need to revise BMI criteria for obesity in Japanese and other Asian populations [54], obesity was defined as a BMI of ≥25 kg/m^2^. According to this definition, we examined 3954 subjects with obesity and 9014 controls for the obesity study, with the control individuals having a BMI of <25 kg/m^2^. Individuals with obesity associated with single gene disorders or with metabolic or endocrinologic diseases were excluded from the study, as were those taking medications that may cause secondary obesity. These subjects were largely the same as those for the BMI study.

Diagnosis of MetS was based on a modified version of the definition proposed by the International Diabetes Federation Task Force on Epidemiology and Prevention; National Heart, Lung, and Blood Institute; American Heart Association; World Heart Federation; International Atherosclerosis Society; and International Association for the Study of Obesity [18]. We used cutoff values for waist circumference of ≥90 cm in men or ≥80 cm in women on the basis of a recommendation of the International Diabetes Association [18]. A total of 3998 subjects with MetS thus had three or more of the following five components: (i) a waist circumference of ≥90 cm for men or ≥80 cm for women; (ii) a serum triglyceride concentration of ≥1.65 mmol/L (150 mg/dL) or drug treatment for elevated triglycerides; (iii) a serum HDL-cholesterol concentration of <1.04 mmol/L (40 mg/dL) for men or <1.30 mmol/L (50 mg/dL) for women; (iv) a systolic blood pressure of ≥130 mmHg, diastolic blood pressure of ≥85 mmHg, or drug treatment for hypertension; and (v) a fasting plasma glucose level of ≥5.50 mmol/L (100 mg/dL) or drug treatment for elevated glucose. History of obesity, dyslipidemia, hypertension, or diabetes mellitus was evaluated with a detailed questionnaire. The control subjects comprised 2819 individuals who had none of the five components of MetS. Autopsy cases were excluded from controls for both the obesity and MetS studies.

The study protocol complied with the Declaration of Helsinki and was approved by the Committees on the Ethics of Human Research of Mie University Graduate School of Medicine, Hirosaki University Graduate School of Medicine, Tokyo Metropolitan Institute of Gerontology, and participating hospitals. Written informed consent was obtained from each participant or families of the deceased subjects.

### EWASs for BMI, obesity, and MetS

Methods for collection and extraction of genomic DNA samples were described previously [53]. The EWASs were performed with the use of the HumanExome-12 v1.1 or v1.2 DNA Analysis BeadChip or Infinium Exome-24 v1.0 BeadChip (Illumina, San Diego, CA, USA). Detailed information of these exome arrays and methods of quality control were described previously [53]. Totals of 41,327 or 41,675 SNPs passed quality control for the BMI and obesity studies and for the MetS study, respectively, and were subjected to analysis.

### Statistical analysis

For analysis of the characteristics of study subjects, quantitative data were compared between subjects with obesity or MetS and controls with the unpaired Student's *t* test. Categorical data were compared between two groups with Fisher's exact test. The relation of genotypes of SNPs to BMI in the EWAS was examined by linear regression analysis. Allele frequencies were estimated by the gene counting method, and Fisher's exact test was used to identify departure from Hardy-Weinberg equilibrium. The relation of allele frequencies of SNPs to obesity or MetS in the EWASs was examined with Fisher's exact test. To compensate for multiple comparisons of genotypes or allele frequencies with BMI, obesity, or MetS, we applied Bonferroni's correction for statistical significance of association. Given that 41,327 or 41,675 SNPs were analyzed in the BMI or obesity and the MetS studies, respectively, the significance level was set at *P* < 1.21 × 10^−6^ (0.05/41,327) or *P* < 1.20 × 10^−6^ (0.05/41,657), respectively. Quantile-quantile plots for *P* values of genotypes in the EWAS for BMI or for those of allele frequencies in the EWASs for obesity or MetS are shown in Supplementary Figure 2. The inflation factor (λ) was 1.03 for BMI, 1.22 for obesity, and 1.13 for MetS. Multivariable logistic regression analysis was performed with obesity or MetS as a dependent variable and independent variables including age, sex (0, woman; 1, man), and genotype of each SNP. A detailed method of analysis was described previously [53]. The relation of genotypes of SNPs identified in the EWASs to BMI or the five components of MetS was examined by one-way ANOVA. Bonferroni's correction was also applied to other statistical analysis as indicated. Statistical tests were performed with JMP Genomics version 6.0 software (SAS Institute, Cary, NC, USA).

## SUPPLEMENTARY MATERIALS FIGURES AND TABLES


