# Supplementary material for: Identification of rs7350481 at chromosome 11q23.3 as a novel susceptibility locus for metabolic syndrome in Japanese individuals by an exome-wide association study
Source: Oncotarget. 2017 Apr 7;8(24):39296–308. doi: 10.18632/oncotarget.16945 (PMC5503614; doi:10.18632/oncotarget.16945)
Supplement: Supplementary file 1 [file oncotarget-08-39296-s001.pdf]

Identification of rs7350481 at chromosome 11q23.3 as a novel susceptibility locus for metabolic syndrome in Japanese individuals by an exome-wide association study

SUPPLEMENTARY MATERIALS

SUPPLEMENTARY FIGURES AND TABLES

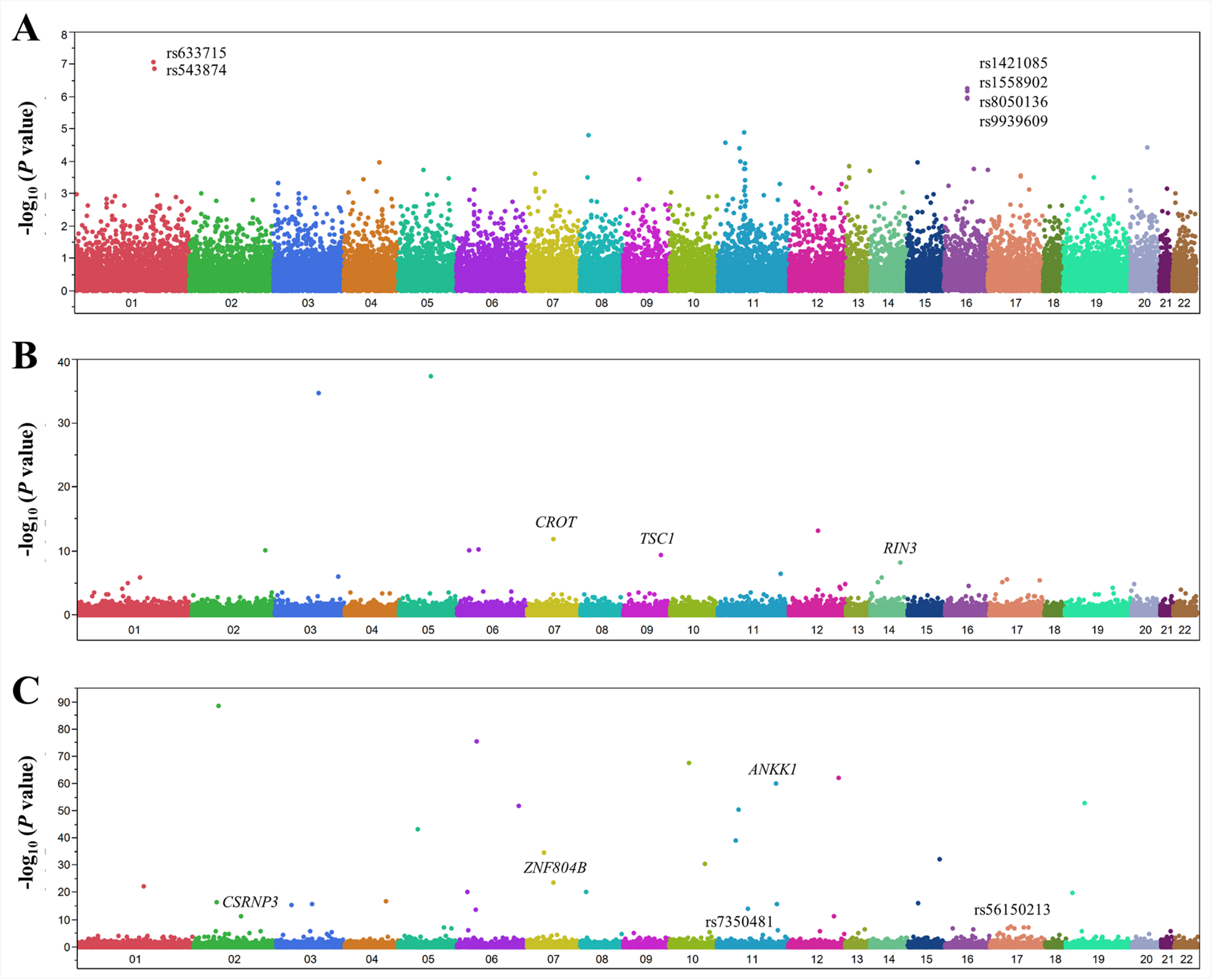

Supplementary Figure 1: Manhattan plots of *P* values in the EWASs for BMI (A), obesity (B), and MetS (C). The *P* values (*y*-axis) are shown as $-\log_{10}(P)$ with respect to the physical chromosomal position of the corresponding SNPs (*x*-axis). SNPs or the corresponding genes identified in the present study are indicated.

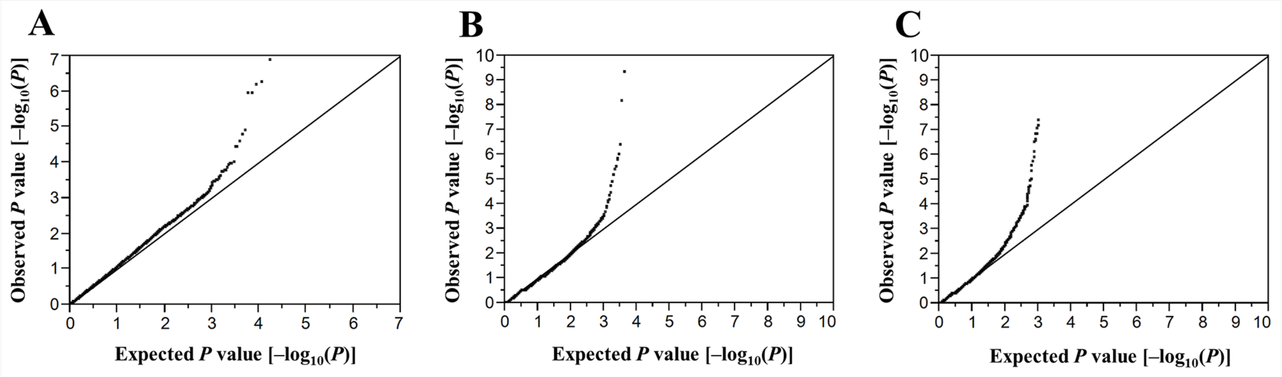

Supplementary Figure 2: Quantile-quantile plots for *P* values in the EWASs of BMI (A), obesity (B), and MetS (C). The observed *P* values (*y*-axis) are compared with the expected *P* values (*x*-axis) under the null hypothesis, with the values being plotted as $-\log_{10}(P)$.

Supplementary Table 1: Genotype distributions for SNPs associated ($P < 1.21 \times 10^{-6}$) with obesity in the EWAS

SNP			Obesity		H-W *P*		Controls		H-W *P*
rs3749745	G/C (E725Q)	GG	GC	CC		GG	GC	CC	
		3820 (96.61)	130 (3.29)	4 (0.10)	0.0307	8665 (96.13)	343 (3.80)	6 (0.07)	0.1651
rs2292661	C/T (T391M)	CC	CT	TT		CC	CT	TT	
		3901 (98.66)	52 (1.32)	1 (0.02)	0.1666	8885 (98.57)	129 (1.43)	0 (0)	1.0000
rs7970885	G/A (V200M)	GG	GA	AA		GG	GA	AA	
		2584 (65.35)	1213 (30.68)	157 (3.97)	0.3322	5876 (65.19)	2827 (31.36)	311 (3.45)	0.2078
rs7808249	G/A	GG	GA	AA		GG	GA	AA	
		1750 (44.26)	1786 (45.17)	418 (10.57)	0.2366	4178 (46.36)	3922 (43.51)	913 (10.13)	0.8843
rs2068204	C/T	CC	CT	TT		CC	CT	TT	
		1568 (39.66)	1837 (46.46)	549 (13.88)	0.7852	3612 (40.07)	4187 (46.45)	1215 (13.48)	0.9819
rs10191097	T/G	TT	TG	GG		TT	TG	GG	
		1988 (50.29)	1638 (41.44)	327 (8.27)	0.6994	4494 (49.86)	3728 (41.36)	792 (8.78)	0.6303
rs757259	G/A (E244K)	GG	GA	AA		GG	GA	AA	
		2801 (70.89)	1056 (26.73)	94 (2.38)	0.6748	6348 (70.46)	2432 (27.00)	229 (2.54)	0.8756
rs1076160	A/G	AA	AG	GG		AA	AG	GG	
		1188 (30.05)	1974 (49.92)	792 (20.03)	0.6072	2630 (29.18)	4427 (49.12)	1956 (21.70)	0.2526
rs8018360	C/T	CC	CT	TT		CC	CT	TT	
		2540 (64.24)	1235 (31.23)	179 (4.53)	0.0674	5829 (64.67)	2850 (31.62)	335 (3.71)	0.5917
rs144079825	G/C (E258Q)	GG	GC	CC		GG	GC	CC	
		3945 (99.77)	9 (0.23)	0 (0)	1.0000	8993 (99.77)	21 (0.23)	0 (0)	1.0000
rs145491613	A/G (N388S)	AA	AG	GG		AA	AG	GG	
		3925 (99.27)	28 (0.71)	1 (0.02)	0.0537	8926 (99.02)	97 (0.97)	1 (0.01)	0.1961

Data are numbers of subjects (percentages). H-W *P*, *P* value for Hardy-Weinberg equilibrium.

Supplementary Table 2: Relation of SNPs to obesity as determined by multivariable logistic regression analysis

SNP		Dominant		Recessive		Additive 1		Additive 2	
		P	**OR (95% CI)**	***P***	**OR (95% CI)**	***P***	**OR (95% CI)**	***P***	**OR (95% CI)**
rs3749745	G/C (E725Q)	0.1972		0.6765		0.1716		0.6819	
rs2292661	C/T (T391M)	0.6186		0.0949		0.5344		0.0950	
rs7970885	G/A (V200M)	0.8568		0.1319		0.5446		0.1573	
rs7808249	G/A	0.0241	1.09 (1.01–1.18)	0.5172		0.0307	1.09 (1.01–1.18)	0.2039	
rs2068204	C/T	0.5791		0.4700		0.7347		0.4257	
rs10191097	T/G	0.6657		0.3958		0.8564		0.3859	
rs757259	G/A (E244K)	0.6263		0.6047		0.7110		0.5816	
rs1076160	A/G	0.2661		0.0340	0.90 (0.82–0.99)	0.6676		0.0410	0.89 (0.80–0.99)
rs8018360	C/T	0.6700		0.0339	1.23 (1.02–1.47)	0.8658		0.0378	1.22 (1.01–1.47)
rs144079825	G/C (E258Q)	0.9551		ND		0.9551		ND	
rs145491613	A/G (N388S)	0.1711		0.5529		0.1429		0.5541	

Multivariable logistic regression analysis was performed with adjustment for age and sex. Based on Bonferroni's correction, a *P* value of <0.0011 (0.05/44) was considered statistically significant. OR, odds ratio; CI, confidence interval; ND, not determined.

Supplementary Table 3: Genotype distributions for SNPs associated ($P < 1.20 \times 10^{-6}$) with MetS in the EWAS.

See Supplementary File 1

Supplementary Table 4: Relation of SNPs to MetS as determined by multivariable logistic regression analysis.

See Supplementary File 1

Supplementary Table 5: Relation of SNPs identified in the present study to phenotypes examined in previous GWASs.

See Supplementary File 1
